# Examining Shape Dependence on Small Mild Steel Specimens during Heating Processes

**DOI:** 10.3390/ma17163912

**Published:** 2024-08-07

**Authors:** Tamás Ibriksz, Gusztáv Fekete, Ferenc Tancsics

**Affiliations:** Department of Material Science and Technology, Audi Hungária Faculty of Vehicle Engineering, Széchenyi István University, H-9026 Győr, Hungary; ibriksz.tamas@ga.sze.hu (T.I.); tancsics@sze.hu (F.T.)

**Keywords:** structural steel, heating time, heat equalization, shape dependence, shape complexity factor, heat transfer

## Abstract

With regard to the heating technology of small test specimens (D < 1 inch, i.e., 25.4 mm), only a limited amount of data and literature are available for making adequate technological decisions. Heating time of small geometric shapes is influenced by the technological parameters of the furnace, the temperature, the disposition technique in the furnace and the geometric characteristics of the workpiece. How to shorten heating time to achieve a suitable material structure is a vital question, while considerable energy is saved at the same time. Among the geometric characteristics, shape dependence is one of the important aspects that must be taken into account in terms of heating technology. Shape dependence is usually taken into account with empirically produced correction factors, which can result in significant oversizing of heating time, energy-wasting technology and material structure of insufficient fineness. In the course of our work, we investigated and compared the shape dependence of cylindrical and prismatic specimens with the same surface-to-volume ratios, which were combined with surface heat transfer analyses and geometric effect tests to formulate new approximate equations for determining heating time. As a result, we could mathematically derive a relationship between heating time, size and shape of the active surfaces, the correlation of which can shorten heating time by 20%. In addition, a shape factor (1.125) between cylinder and prismatic-shaped specimens was determined, which can be used with the new equation to calculate heating time for similar specimens. At last, a relationship is developed between the amount of heat that can be stored in the body during heat equalization and the complexity of the shape, which can be characterized through ratios depending on heating times and active surfaces in the function of total surface/volume ratio. Based on this relationship it can be determined more precisely when heat equalization occurs; therefore, shorter heating time can be achieved. In conclusion, with the help of this new method, optimal heating time for structural steel components, in the case of small cross-section and weight, can be determined.

## 1. Introduction

During the design process of a heat treatment technology, several key parameters must be determined. Such key parameters are furnace performance, heating positions, heating time and heating speed. The latter two parameters can be significantly influenced by material quality, material structure and the geometry of the component. Several studies were carried out taking different test objectives into account to ensure material quality and material structure by a given heat treatment technology. Most of them drew attention to the relationship between heat treatment technology and the produced material structure after heat treatment.

Such areas can be the quality and fineness of the grain structure [1,2], the influence of precipitates and transformations [3] or the manufacturability conditions of certain required material structures [4,5,6]. Only a negligible part of the studies deals with the geometrical properties of the components and the heating conditions that can be derived from them [7]. These studies mostly focus on size effects and are usually developed for characteristic size ranges larger than 1 inch [8].

Other geometric properties, such as the complexity of the shape or the segmentation of the geometry, are mostly taken into account with correction factors [9]. At the same time, the experimental determination of correction factors is a costly task, even on an industrial scale. In addition, it does not always lead to satisfactory results, especially in the size range smaller than 1 inch.

The majority of automotive and structural parts sizes can be classified in the geometric range of less than 1 inch. These parts are mostly made from HSLA steel. Accordingly, S460N-type structural steel, weakly alloyed with manganese (Mn), was used for our experiments. It must be noted that steels with Mn alloying elements are more sensitive to the heating process due to the risk of unfavorable grain coarsening [10].

Common but valid phase that heat treatment technologies are extremely diverse and, in most cases, they must be adapted to a specific product. As a result, there is no recognized guiding principle for the exact determination of heating time, only approximate, empirical solutions, which fundamentally affect the quality of the heat treatment technology. 

Determination of the heating time, even nowadays, is mostly based on empirical relationships. It is worth noting that several disadvantages can be credited to unnecessarily prolonged heating time, e.g., coarse material structure and high energy waste.

In this work, optimal heating time for geometries smaller than 1 inch is investigated in order to create a mathematical relationship between the surface heat transfer processes and the characteristics of the shape complexity. Heating time is considered acceptable up to the point until complete heat equalization occurs in the material. The process of heat equalization was examined in some scientific works with regard to high-temperature heat transfer processes [11,12]; however, the nature and utilization of heat transport processes were not analyzed in detail. Nevertheless, these observations are important to us since during our experiments, heat transport processes of a similar nature are expected to take place over a relatively short period of time. It is assumed that the characteristics of heat diffusion processes can be generalized in certain geometric ranges as a function of heat transfer on the surfaces.

With the shape dependence of heat transport, in the case of a given geometry, heat treatment parameters can be more precisely determined. Since shape dependence is usually taken into account with the shape complexity factor, we do not want to deviate from this in our work. The primary objective of our experiments is to examine the shape dependence of the heating process, as well as the effect of different shapes on the surface heat transfer coefficient and the heating time. These geometries are elementary forms (cylinder, prism) from which a simple component can be built. In the course of our experiments, we would also like to define a system of connections, which could create free passage between individual shape complexities.

## 2. Materials and Methods

In our experimental investigations, heating and cooling experiments on specimens with different cross-sections (circle and square) but the same surface-to-volume ratio (0.233 mm^−1^) were performed. The main dimensions of the specimens were Ø20 × 60 mm in the case of cylindrical geometry, and mostly 20.75 × 20.75 × 49.3 mm in the case of prismatic geometry. For each test, 5 specimens with each geometry type were used. Heating data were collected on all specimens to select the one containing the least error and noise.

### 2.1. Data Collection

The material quality of the specimen met the requirements of S460N structural steel. The test specimens were cut from the columnar crystalline zone of a continuously cast block with a cross section of 100 × 100 mm. The grain structure of the test specimens consisted of fine ferrite and pearlite, and its grain fineness corresponded to the ASTM Standard [13] 10–11 grain size class. The carbon equivalent (CE according to EN 1011-2/A) [14] is 0.483, which ensures excellent weldability. The S460N material grade, which is used for the heating experiments, slightly differed from the standard average. The most important alloying elements were present as follows: C = 0.141%, Mn = 1.5%, Ni = 0.62%, Si = 0.18%, Al = 0.008%, Cu = 0.2%, Ti = 0.0067%, and V = 0.186%.

**Figure 1:**
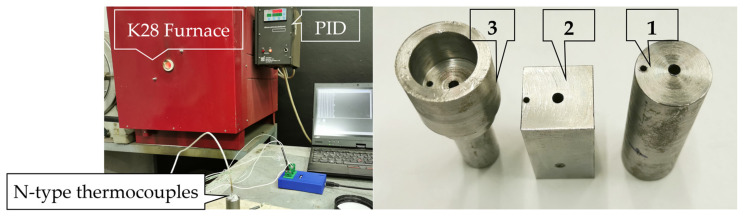
The assembled measurement system (left), the cylindrical (1), the prism-shaped (2) and the more complicated test specimen (3).

The heating experiments were carried out in an electric resistance-heating furnace type of K-28/1100 (Kalória Hőtechnikai Kft, Budapest, Hungary), pre-set to a temperature on 1060 °C. The constant temperature of the furnace space was ensured by a PID (Proportional Integral Derivative) temperature control system, while forced convection was not used. Thermocouples were placed directly below the surface (hereinafter this will be considered the surface temperature) and along the axis of the test bodies to observe the process of heat equalization. When determining the heat transfers, we did not consider the minimal heat loss associated with placing the specimen in position due to the fast correction of the PID system. Figure 1 shows the measurement system and the test specimens.

**Figure 2:**
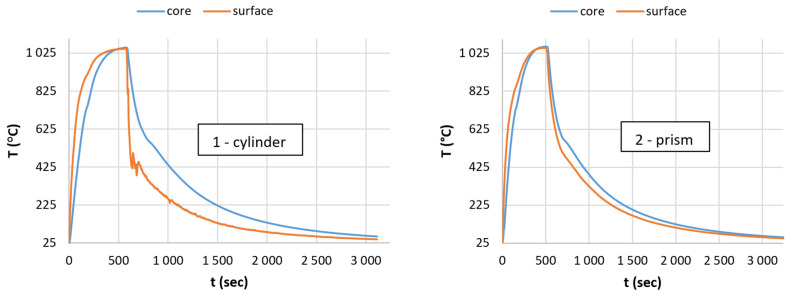
Temperature change on the surface and in the axis line of the test specimen in the case of cylinder and prismatic shapes.

During the experiments, we attempted to heat the test specimens under the same conditions until they reached thermal equilibrium. Thereafter, the temperature was maintained for a short time to achieve the moment of heat equalization (approaching the 0 value of holding time). This step was followed by the cooling with air. Measurement results can be seen in the temperature-time diagrams in Figure 2.

**Figure 3:**
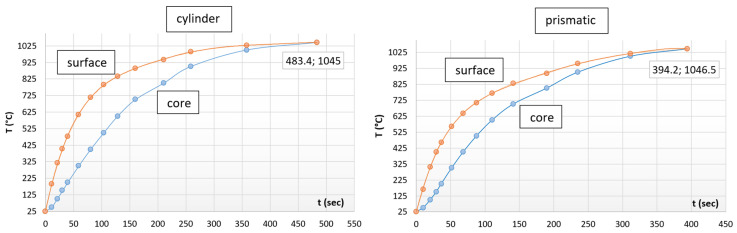
Representation of data suitable for analysis in the case of cylindrical and prismatic specimens.

In the first step, we reduced the data to a manageable amount by a suitable algorithm and continued to work with the diagrams shown in Figure 3. The measurement noises caused by electrical contact disturbances can be smoothed out by fitting polynomial curves where necessary.

Figure 3 clearly illustrates the difference in heating efficiency in the case of cylindrical and prismatic specimens. It seems evident that during heating the heat equalization process between the surface and the core is more effective for the prismatic-shaped test specimens. However, we assumed that there is a limit where the surface-to-volume ratio reverses. In order to establish this limit, we also examined an entire interval characterized by side dimensions ranging from a = 20 × 20 mm to a = 40 × 40 mm (with the same surface-to-volume ratio). The size limits of the prismatic-shaped specimens were determined by the manageability in the stand state on the squared surface. We found that the prismatic-shaped specimens, compared to the active surface of the cylinder, have a larger active surface up to a size of a = 21.5 × 21.5 mm. At this size limit, the relationship reverses, while above this limit the relationship shows no more change within the examined interval. 

This means that the active surface of the Ø20 × 60 mm cylinder, in the stand state, is 4082 mm^2^, while the active surface of the prismatic specimen closest to it (21.5 × 21.5 × 42.3 mm) in the stand state is 4099.63 mm^2^. Therefore, in the examined interval, in the same position, above the side size of 21.5 × 21.5 mm, the following active surfaces will be smaller than 4082 mm^2^. This finding will be important later.

### 2.2. The Biot Number

Before we proceed further to the examination of the relationships, it is necessary to establish the ratio of the thermal resistances determining the heat transfer process on the surface and at the axis line of the specimen. Depending on the results of the test, we are faced with either surface heat transfer or heat conduction issues depending on which property becomes more dominant in terms of heat spreading. 

The Biot number is a suitable method to decide the method of heat spreading [15]. If *Bi* < 0.1, the heat flow is determined by the surface heat transfer processes, which is the heat transfer ability of the surface. The relation used for the definition:

(1) $$Bi=\frac{\alpha_{EHTC}}{\kappa_{E}}\cdot L^{\prime }$$

where Bi is the dimensionless Biot number (-), $\alpha_{EHTC}$ is the effective heat transfer coefficient (sum up convection and radiation) (W/m^2^·K), $\kappa_{E}\;$ is the effective thermal conductivity of scaled specimen (W/m·K) and $L^{\prime }\;$ is the characteristic length (ratio of the volume and the surface) (m).

It is evident that the prismatic-shaped specimens have a larger active surface than the cylindrical specimens with the same surface-to-volume ratio. Earlier, we established that this relationship is true only up to a certain size limit, which will also mark the validity limit of our primary tests. For both geometries, the heating was carried out one by one in the vertical position during the experiment. The covered surfaces were not taken into consideration. Accordingly, we defined the Biot number in the case of the cylindrical specimen giving the highest heating time value. To determine the effective heat transfer coefficient, which can be found in relation (1), the following equation was taken into account which is obtained by the linear superposition of heat fluxes [16]:

(2) $$\alpha_{EHTC}=\alpha_{R}+\alpha_{C}$$

where $\alpha_{R}$ is the radiation heat transfer coefficient (W/m^2^·K) and $\alpha_{C}$ is the convective heat transfer coefficient (W/m^2^·K).

In the following step, we had to consider that heat transfer basically takes place by radiation if an electric-resistance heating furnace is used. It must be noted that natural convection around the specimen is an order of magnitude smaller and it is usually characterized by empirical values (e.g., 15 W/m^2^·K) [16]. 

It also follows from Equation (2) that the surface convection heat flux density (q_C_) and the radiation heat flux density (q_R_) sum up. The radiation heat flux density is determined by the difference between the absorbed radiation and the emitted radiation from the surface to the heat equilibrium state of the test object.

In the latter case, the heat flow passing through the surface can be neglected. 

The following thermal radiation coefficient can be derived from the equilibrium state (Kirchhoff’s law), which suitably sums up small temperature ranges [17]:

(3) $$\alpha_{R}=\varepsilon \cdot \sigma \cdot \left(T_{f}+T_{S}\right)\cdot \left(T_{f}^{2}+T_{S}^{2}\right)$$

where ε is dimensionless emissivity constant (henceforth, its value is 0.8), σ is the Stefan–Boltzmann constant (5.67 × 10^−8^ W/m^2^·K^4^), $T_{f}\;$ is the furnace space temperature inside (K), $T_{S\;}$ is the surface temperature of the specimen (K) and $\alpha_{R}\;$ is the radiation heat transfer coefficient (W/m^2^·K).

Solving Formula (3) provides a good approximation of the heat transfer coefficient characteristic of heat radiation. Before solving the relation, we assume that the temperature of the air is the same as the temperature of the furnace space if a sufficient distance is considered. The effect of the convection heat transfer coefficient is practically negligible; however, for the sake of certainty, it was determined by the use of the Nusselt number [18]. The Nusselt number takes into account the geometrical shape, the position of the specimen and the flow nature around the specimen during the convection heat exchange.

The Nusselt number is defined as a ratio between the convective and the conductive heat transfer:

(4) $$Nu=\frac{\alpha_{C}\cdot L}{\kappa_{air}}$$

where Nu is the dimensionless Nusselt number (-), *L* is the crucial length of the specimen (m) and $\kappa_{air}\;$ is the effective thermal conductivity of the air film near the surface (W/m·K). Rearranging Equation (4), the convection heat transfer coefficient can be expressed as (5):

(5) $$\alpha_{C}=\frac{Nu\cdot \kappa_{air}}{L}$$

The Nusselt number hence considers the position and the shape of the specimen in the case of natural convection as well. Therefore, the cylinder in a fixed position was expressed by (6) [19] and the prismatic-shaped specimen in a fixed position was expressed by (7) [20] equations.

(6) $$Nu=-0.3903+0.5399\cdot \sqrt[{4}]{Ra}+0.6367\cdot \frac{L}{D}\;\;\;if\;2\leq \frac{L}{D}\leq 10$$

(7) $$Nu=0.59\cdot \sqrt[{4}]{Ra}$$

where Ra is the dimensionless Rayleigh number (-) and *D* is the diameter of the specimen (m).

The Rayleigh number can be used to determine when the airflow on the surface of the test specimen becomes turbulent. If this number is greater than 10^9^, the flow is turbulent. This value remained in the range of 10^5^ during our experiments, so a laminar airflow surrounded the test specimens. This number can be obtained by multiplying the Prandtl (*Pr*) and Grashof (*Gr*) numbers. 

Both numbers are dimensionless numbers. The relations (8) and (9) were used to determine the *Pr* and *Gr* numbers.

(8) $$\mathrm{Pr}=\frac{\mu \cdot {c_{p}}_{\left(air\right)}}{\kappa_{air}}$$

where Pr is the dimensionless Prandtl number (-), μ is the dynamic viscosity of dry air near the specimen surface (kg/m·s), $c_{p(air)}\;$ is the specific heat of dry air near the specimen surface (J/kg·K) and $\kappa_{air}\;$ is the thermal conductivity of dry air near the specimen surface (W/m·K).

(9) $$Gr=\frac{g\cdot L^{3}\cdot \beta \cdot \left(T_{air}-T_{S}\right)}{\nu^{2}}$$

where g is the gravitational acceleration (9.81 m/s^2^), β is the dry air thermal expansion coefficient. A good approximation for gases is the reciprocal of the air temperature in Kelvin: (1/K). $T_{air}\;$ is the dry air film temperature that surrounds the specimen, which is the average of the surface temperature of the furnace space and the test specimen (K). *ν* is the kinematic viscosity of dry air near the specimen surface (m^2^/s).

Using the method presented above, we determined the effective heat transfer coefficients and their components at temperature intervals of 50 to 100 °C. The average heat transfer coefficient values obtained in this way were as follows: *α_EHTC_* = 299.3 W/m^2^K for the cylindrical specimen and *α_EHTC_* = 286.5 W/m^2^K for the prismatic-shaped specimen. All in all, a small difference of 12.8 W/m^2^K (4.3%) between the two specimens can be found.

Returning to relation (1), it is still necessary to clarify the thermal conductivity of the test specimens in order to determine the Biot number. In our previous studies, we have already dealt with the topic of scaling and found that during our experiments, the formed scale thickness can be approximated well according to the modified J. Païdassi correlation [21]. The boundary conditions for this heating technology: S460N steel quality, the usage of an electric-resistance heating furnace and specimens set in vertical position one by one. We also used this relationship during our present experiments:

(10) $$X=24550\cdot \exp-\left(\frac{84650}{RT}\right)\cdot \sqrt{2t}$$

where X is the scale thickness at a given moment (μm), R is the universal gas constant (8.314 J/K·mol), T is the heating temperature which is equal to the surface temperature (K) and t is the heating time of specimen (s).

**Figure 4:**
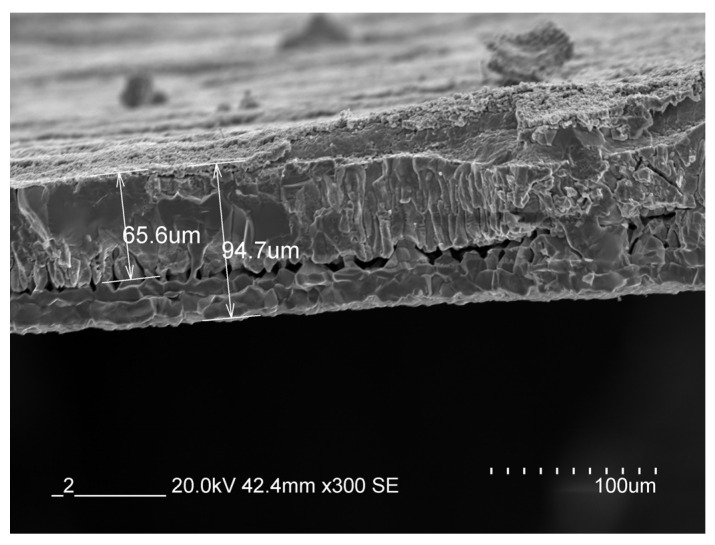
The measured thicknesses of scale using SEM-EDX: Hitachi S3400N (Hitachi, Berkshire, UK) and Bruker EDX detector (Bruker, Vienna, Austria).

Based on relation (10), the expected scale thickness at a temperature of 1045 degrees Celsius during the experimental heating time (483.4 s) is 95.1 microns. This is close to the average value of our measurements, which is illustrated in Figure 4.

The thickness of the scale is negligible compared to the cross-section of the test specimen, but its thermal conductivity is significantly lower, so it cannot be ignored [22]. The specific thermal conductivity of S460N steel varies with temperature, while its average value is 27.17 W/m·K. The thermal conductivity of scale is an order of magnitude lower than with the structural steels. Therefore, taking the literature references into account, the specific thermal conductivity of scale was taken as 2.5 W/m·K [23,24,25]. 

Finally, we determined the probable average thermal conductivity of the cylindrical specimen in proportion to the thicknesses, where the value corresponded to 26.94 W/m·K. Based on the above, the Biot number can be determined. The value of the Biot number is 0.048, which from the point of view of the experiment, means that the surface heat transfer plays a decisive role in the thermal processes taking place during heating.

## 3. Results

### 3.1. The Heat Transfer Factors

**Figure 5:**
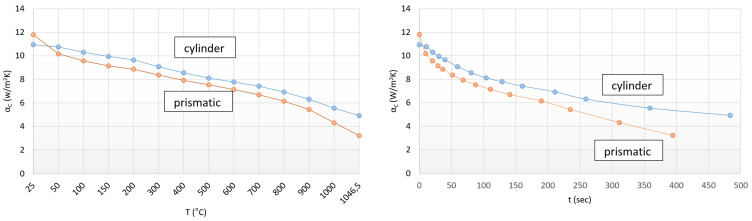
Trends of convective heat transfers in the case of cylindrical (D20 × 60 mm) and prismatic-shaped (20.75 × 20.75 × 49.3) specimens.

In the following step, we investigated the detectability of the surface complexity based on the evolution of the heat transfer factors. The diagrams shown in Figure 5 can be drawn in relation to the convection heat transfer coefficients.

The curves in Figure 5 show that a smaller heat transfer coefficient is required to reach the same temperature in the case of the specimen with a larger active surface (Figure 5, left side). Both curves on the left side can be well approximated by a fourth-degree polynomial. 

The inflection points of these polynomials can be determined. Beyond these temperature points the convection, which surrounds the surface of the specimen, gradually stops. 

Therefore, in both cases, the downward trend of the convection heat transfer factor can be seen with increasing temperature. Thus, based on the curves it can be concluded that a shorter heating time is required in the case of a larger active surface to complete heat equalization (Figure 5, right side).

Based on the curves, we can make a preliminary statement as well. In the case of the same surface-to-volume ratio, the sample with smaller convection heat transfer values can be heated in a shorter time, besides approximately the same intensity and an order of magnitude higher heat transfer of radiation. The average convection heat transfer coefficient was 8.3 W/m^2^K for the cylindrical sample and 7.6 W/m^2^K for the prismatic-shaped sample. The convection heat transfer of the prismatic at the start and end points is apparently different from the parallel run characteristic of the two curves, which is due to the corner effect.

**Figure 6:**
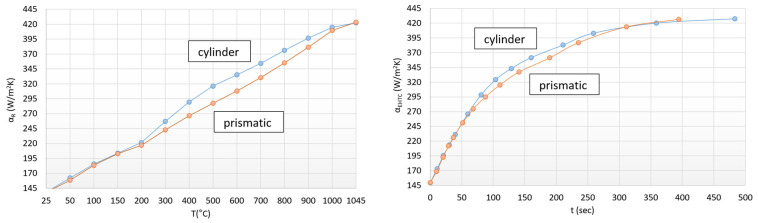
Trends of aggregated heat transfers in the case of cylindrical and prismatic-shaped specimens.

Figure 6 shows the summarized change in the convection and radiation heat transfer coefficient.

The curves clearly show the determining nature of heat transfer by radiation. It is clear that the radiant heat transfer numbers, which are an order of magnitude higher, override the convection heat transfer numbers. In Figure 6, the aggregated heat transfer coefficients of the cylindrical (marked in blue) and prismatic-shaped (marked in orange) specimens can be seen as a function of temperature and time. Based on the curves, it can be established that in the case of the same surface-to-volume ratio, the sample with smaller aggregated heat transfer values can be heated in a shorter time. It can be also derived from these results that a lower heat transfer factor indicates a more efficient heat transfer process due to the larger active surface.

This phenomenon cannot be explained by the different thicknesses of the scale in the case of the tested specimens. The formation time of the scale beside the similar heating technology is primarily determined by temperature, composition of the steel, time, oxygen partial pressure, and the oxidizing environment, which is the same in both cases except for heating time. 

The only difference is the heating time which depends on the complexity of the shapes. Logically, we can consider that a shape that has a larger overall surface is more complicated. In our case, this is the prismatic-shaped specimen. Thus, the different scale thickness may occur only due to the difference in heating times. It can be also derived from this conception that the heating time of more complicated shapes decreases in proportion to the size of their surface.

**Figure 7:**
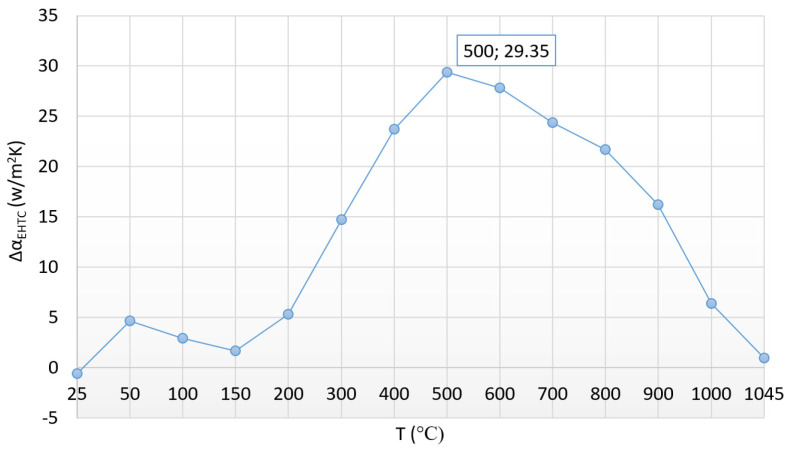
The process of heat equalization from the point of view of the difference in the aggregated heat transfer coefficients during the heating.

Continuing this line of thought, we also compared the aggregated heat transfer factors as a function of temperature as well. A deviation of 1.5 °C of the heat equalization (cylinder heating time versus prismatic-shaped specimen heating time) was ignored. The result is shown in Figure 7.

Figure 7 shows the difference between the aggregated heat transfer coefficients of the prism-shaped and cylindrical specimens as a function of temperature. 

Interpreting Figure 7, it becomes easy to understand the difference in heat transfer resulting from the different shape complexity of the prismatic test specimen and the cylindrical test specimen. 

The curve shows, in accordance with the curves in Figure 6, that up to a temperature of 200 °C, apart from a small fluctuation, the heat transfer takes place similarly for both test specimens. Above 200 °C, however, the intensity of the heat transfer is significantly different. The sharp increase in the differences between the heat transfer factors can be associated with the prominence of the differences between the geometries. The extreme value of the curve shows the highest difference at 500 °C, which balances out as the temperature increases further, taking practically the same value for both specimens. The extreme value of the difference in heat transfer factors indicates that heat propagation in the prism-shaped specimen is most intensive at this temperature due to the larger active surface of the specimen. 

### 3.2. The Heat Equalization Process

**Figure 8:**
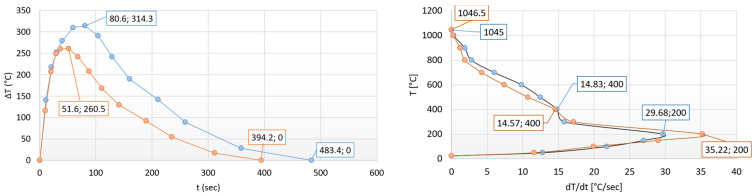
The heat equalization trends of the cylindrical (blue curve) and prism-shaped (orange curve) specimens during heating.

Figure 8 shows the heat equalization and the equalization rate curves (heat-flow rate) of the cylindrical (blue curve) and the prism-shaped (orange curve) test specimen as a function of heating time and temperature. In both cases, the same characteristic curves with similar maximums can be observed. 

Based on the details on the left side of Figure 8, it can be established that the temperature profile curve of the prism-shaped test specimen runs parallel to the profile curve of the cylinder test specimen on a smaller delta *T* value. The relationship is given by the difference in Biot numbers (at the cylindrical specimen is higher) and the intensity difference in surface heat transfer processes. All these deviations are due to the differences in the shape complexity. Based on the course of the temperature profile curves, we can conclude a statement. In the case of structural steels, in the size range of *D* < 1 inch, the profile curves show a significant similarity with the temperature profile curves with a large cross-section of austenitic stainless steel (STS) [11]. The reason for the high degree of similarity is probably the negligible amount of latent heat operating in the case of small cross-sections.

On the right-hand part of Figure 8, it can be seen that the heat flow reaches its highest speed for both geometries at a temperature of 200 °C. With the prism, this rate is significantly higher due to the geometric advantages (larger surface area). As the temperature increases, the speed of the heat flow (heat diffusion) decreases and stops around 1045 °C due to the thermal equalization.

The breaking points of the curves at a temperature of 400 °C refer to the equalization of the heat flow rates, after which the rate of heat equalization is uniform as temperature increases.

### 3.3. The Optimal Heating Time

Based on these results, it can be concluded that in the case of specimens with the same surface-to-volume ratio, there is a relationship between heating time, the amount of absorbed heat and the complexity of the surface. This connection assumes that the amount of heat required to heat up the mass of the specimen is equal to the sum of the heat in-flowing in the surface of the specimen and the amount of heat equalized in the core. This phenomenon can be expressed with the help of surface and time ratios as well. 

To describe this relationship, we also assume that the cylinder can be characterized by a height dimension and an area designated by the perpendicular distance between the surface and the core, which, when rotated 360 degrees around its centerline, produces a cylinder. The relationship derived based on the above can be written in the following form (11):

(11) $$\frac{\frac{c_{p}\cdot p}{t_{C}}\cdot x^{2}\pi -\lambda }{\alpha_{EHTC(c)}\cdot x^{2}\pi }\cdot x=\frac{A}{V}\cdot x≅\frac{t_{C}}{t_{P}}+\frac{A_{P}}{A_{C}}=2.33$$

where $c_{p}$ is the specific heat of the specimen considering the scaled surface at 1045 °C (611.585 J/kg °C), p is the density of the specimen considering the scaled surface at 1045 °C (7481.43 kg/m^3^), x is the perpendicular distance between the surface and the core referring to the radius of cylinder (m), λ is the heat conduction of S460N steel at 1050 °C (−28.35 W/m °C), $\alpha_{EHTC(c)}$ is the aggregated heat transfer coefficient of the cylindrical specimen at 1045 °C (427.5 W/m^2^ °C), A/V is the value of the entire surface-to-volume ratio expressed in meters (m^−1^), $t_{C}\;$ is all heating time of the cylindrical-shaped specimen (s), $t_{P}\;$ is all heating time of the prismatic-shaped specimen (s), $A_{P}\;$ is the active surface of the prismatic-shaped specimen (m^2^) and $A_{C}\;$ is the active surface of the cylindrical specimen (m^2^). 

**Figure 9:**
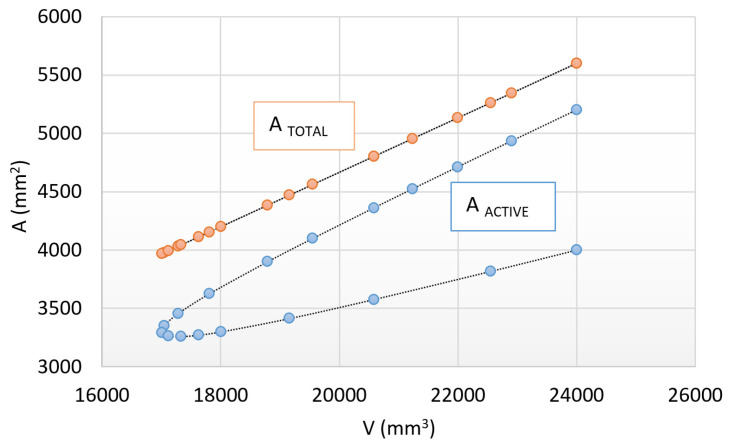
Interpretation of fulfillment of the surface/volume ratio.

Based on relation (11), an unknown parameter of a cylindrical or prismatic specimen with the same surface-to-volume ratio, which can primarily be the heating time, can be determined. To check the suitability of the relationship, we determined again the heating time of the prismatic specimen using the new formula based on our previous experimental data. Based on the new formula *t_C_* = 394.5 s, which proves to be sufficiently accurate compared to the total heating time of the prism-shaped test piece (394.2 s). Therefore, we believe that relation (11) can be extended to the entire size range defined earlier (from *a* = 20 × 20 mm to *a* = 40 × 40 mm) as well. In order to do this, we must first ensure the same proportions of total surfaces and volumes, which can be seen in Figure 9.

Figure 9 points out that the previously determined surface-to-volume ratio is achieved in the case of a full surface; however, it is not fulfilled in the case of active surfaces. Within the same system, this difference will be the main driver of the difference in heating times. To provide an answer to this problem, a reference surface ratio of the prismatic specimen should be created, while the cylinder temperature equalization time is preserved (12).

(12) $$\frac{A}{V}\cdot x=\frac{t_{C}}{t_{PN}}+\frac{A_{P}}{A_{PN}}$$

where $t_{C}$ is the complete heating time of the cylindrical-shaped specimen (s), $t_{PN}\;$ is the complete heating time of the new prismatic-shaped specimen (s), $A_{P}\;$ is the active surface of the prismatic-shaped specimen, which is the reference data hereafter (m^2^) and $A_{PN}\;$ is the active surface of the new prismatic-shaped specimen (m^2^)

On the basis of relation (12), the theoretical heating time data can be determined for the entire size range (from *a* = 20 mm to *a* = 40 mm). However, due to the surface ratio of the reference prismatic specimen (the new reference ratio is 1, since *A_P_* = *A_PN_*), smaller values can be obtained compared to the real values. The new reference value is *t_PN_* = 362.55 s. This deviation must be corrected with a correction ratio (1.088) to the value calculated with relation (11) in order to obtain the realistic values: *t_PN_* = 394.45 s. With this correction, we made the relationship suitable for examination on the entire size range of the prismatic-shaped specimens. Heating time over the entire range can already be approximated well with a third-degree polynomial (*R*^2^ = 0.9988). The equation of the third-degree polynomial according to relation (13):

(13) $$t_{PN}=0.11a^{3}-11.417a^{2}+381.06a-3578.7$$

After rechecking the heating time of the reference prism-shaped test piece by applying relation (13), we obtain 395.3 s, which is an acceptable difference compared to the originally measured 394.2 s. The difference is only 1.1 s.

Next, it is worth examining the extreme values, i.e., the heating time of the test specimens with *a* = 20 mm and *a* = 40 mm side distances. The total surface area of these test specimens is individually 5600 mm^2^, their volume is also the same, 24,000 mm^3^, and their surface-to-volume ratio is also the same (Figure 9). Only their active surfaces differ in terms of heat transfer. The active surface of the prismatic-shaped specimen with *a* = 20 mm side distance is 5200 mm^2^, and the active surface of the prismatic-shaped specimen with *a* = 40 mm side distance is 4000 mm^2^. 

According to our expectations, the heating time of the test specimen with *a* = 20 mm side distance could be shorter. According to the third-degree polynomial, the heating time is 355.7 s for the prismatic-shaped specimen with *a* = 20 mm side distance, while it is 436.5 s for the prismatic-shaped specimen with *a* = 40 mm side distance. 

In addition, it may be interesting to determine the heating time of the minimum active surface within the given range, with the same surface-to-volume ratio, since this is where the extreme value of the polynomial can be found. 

The extreme value of the polynomial is regarded to the sample with *a* = 28 mm side distance, where the heating time is 554.8 s. This is the maximum heating time in the range. Based on the results of the above tests, boundary conditions for prismatic shapes can be formulated as follows:

- The nature of heat transfer must be similar to the above-investigated system; consequently, only the size of the active surfaces can affect the required heating time.
- $0.3<\frac{H}{D_{\left(average\right)}}<3$.

**Figure 10:**
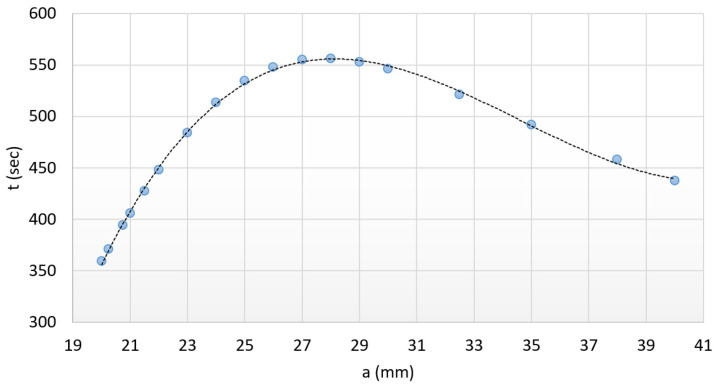
Representation and approximation of heating times with a third-degree polynomial.

It is worth returning to the earlier established theory with regard to the active surface of the prismatic-shaped specimen and cylinder. The active surface of the D20 × 60 mm standing cylinder can be closely approximated by the active surface of the 21.5 × 21.5 × 42.3 mm standing prismatic-shaped specimen. With the same active surface and the appropriate *H*/*D* ratio, the heating time can be calculated according to Equation (13) as 429.8 s. The difference in the same heating conditions is only caused by the difference in shape of the geometries. This ratio can be determined, which is 1.125. Therefore, if the heating time of a prismatic-shaped specimen with the same active surface is corrected by the earlier-mentioned factor (429.8 × 1.125) then 482.625 s is obtained, which is close to the measured value of 483.4 s. Accordingly, with this method, the shape factor between the two different geometries could be determined. Figure 10, as a summary of the above, shows the graphical display of relation (13).

### 3.4. The Applicability of Heating Time

**Figure 11:**
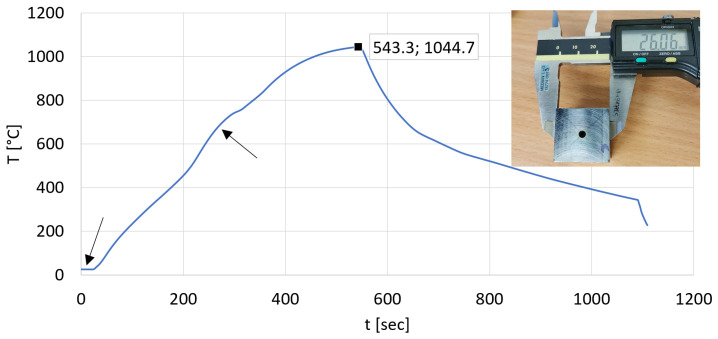
Checking the adequacy of the three-degree polynomial by experiment.

The applicability of the curve shown in Figure 10 was also tested in practice. Before heating, we verified the dimensions of the test piece of 26 × 26 mm side distanced with 25.2 mm height by simple measurement. According to Figure 11, the measurable distance between the flat sides is 26.06 mm referring to 26 mm; therefore, this difference is negligible. 

Based on the diagram in Figure 10 and Equation (13), the heating time of the prismatic test specimen with a side distance of 26 × 26 mm and the same surface-to-volume ratio was determined as 544.328 s. The heating time verified by measurement based on Figure 11 is 543.3 s. The difference is 1.028 s. Practically identical results were obtained. 

In Figure 11, measurement difficulties are depicted with arrows. The first arrow shows the inertia of the thermocouple and the second shows the feedback correction, which ultimately can lead to an appreciable measurement result. Due to the measurement difficulties, it can only be concluded that the result of the measurement is closely similar to the calculated value. At the same time, it can be rightly assumed that a thermocouple, which works more precisely, will be able to justify the calculations.

The reliability of the measurement results, recorded during the experiments, was checked by Simufact Forming FEA analysis. The governing equation of the heat transfer analysis in the applied procedure (14):

(14) $$C\dot{\vec{T}}+K\vec{T}=\vec{Q}$$

where C is the heat capacity matrix (J/K), $\dot{\vec{T}}\;$ is the time derivative vector of the temperature (K/s), K  is the thermal conductivity matrix (W/m·K), $\vec{T}$ is the nodal temperature vector (m·K) and $\vec{Q}\;$ is the thermal load vector (W). In our case, Equation (14) can be reduced to the steady-state problem, since heat transfer is constant in time (15):

(15) $$K\vec{T}=\vec{Q}$$

**Figure 12:**
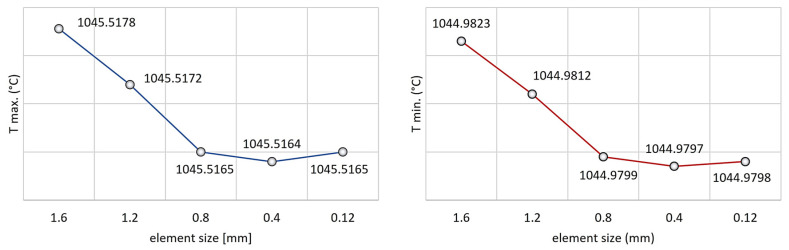
The results of the mesh independence tests.

In order to achieve the finalized results, several analyses were performed to optimize the element size. The element size was considered optimal when it met the conditions of meshing independence. The test was carried out for the maximum and minimum temperatures as well. The results can be seen in Figure 12.

Independent element size was determined as 0.8 mm (see Figure 12). Simufact Forming proposes two kinds of meshing strategies and three different element types to solve 2D meshing problems. In our case, the Quadtree meshing was chosen as a suitable strategy for 2D heat flow modeling. This meshing strategy starts meshing the workpiece from inside to outside with regular quads in the direction of the main axes. Thereafter, the software meshes the gaps, near the corners, based on the allowed element distortion. In accordance with this strategy, we chose the element type with plane strain formulation. In this regard, it can be assumed that strain in depth direction is not possible.

During the analysis, we determined the temperature of the furnace space at which heat equalization between the surface and the core is established. This was based on the determined heating data, the summed heat transfer coefficient and heating time. For the test, we used the data of the cylindrical specimen. The heating data used for the simulation can be classified as acceptable if the virtual temperature of the furnace space is within the sum of the permissible error limits for the thermocouple and the PID temperature controller of the furnace. This cumulative margin of error at a temperature of 1060 °C is defined as follows:

- Thermocouple made by TC Direct (Uxbridge, UK), N type, 1.5 mm and 3 mm diameter, Class 2: at a temperature of 1060 °C, the permissible deviation is 8 °C.
- Furnace space PID control unit: permissible deviation at a temperature of 1060 °C is 10.6 °C.

For this reason, the allowable deviation of the temperature field, at a temperature of 1060 °C, is 1.75%. Input data for the FEA model can be found in Table 1.

**Figure 13:**
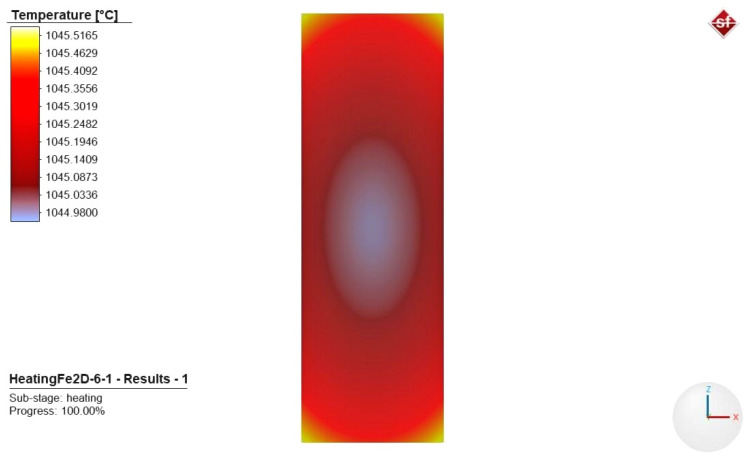
The heat equalization result is in the case of 1046.5 °C of furnace space temperature.

Based on the FEA test, the furnace space temperature can be well approached by the value of 1046.5 °C, which is shown in Figure 13. This value differs by only 1.25% from the set temperature of 1060 °C.

Based on Figure 13, a temperature difference of 0.5365 °C can be established between the core and the surface, which is negligible in the case of the given geometry. In addition, based on the results of the above analysis, the deviation of 1.25% value in temperature can be classified as acceptable for heat treatment exercises.

## 4. Discussion

It is an open question whether heating time can be approximated if a more complex shape, with a different surface-to-volume ratio, is considered. For complex shapes, the same geometry and position cannot always be ensured in the furnace. Therefore, in the case of comparative analyses, these must be adapted to the theoretical basic forms and surface system used in the test system.

**Figure 14:**
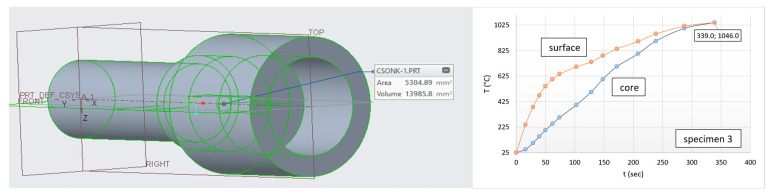
Shape with complex geometry (spindle model) and its heating process.

Figure 14 shows a shape with a more complicated geometry, with a larger active surface than before. The right part of the figure shows the process of heat equalization during heating with the final values. Moreover, the spindle model has a different surface-to-volume ratio (379 m^−1^) as well.

**Figure 15:**
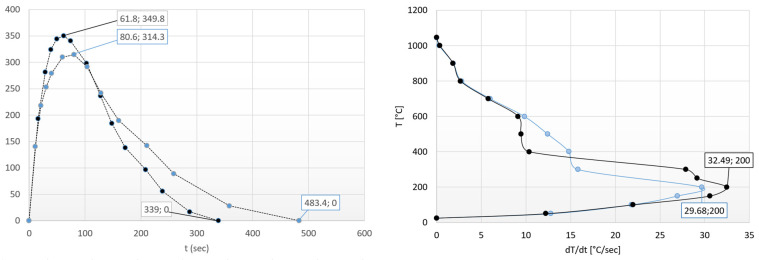
Heat equalization trends of the different complexity specimens.

After the heating experiments, the heat equalization and its rate of equalization curve of the new geometric shape can also be plotted as a function of heating time and temperature (see Figure 15). In Figure 15, the thermal equalizing curve of the former cylindrical (80.6 s/314.3 °C) specimen can be seen with light blue dots, while dark blue dots denote the equalizing curve characteristic of the new shape (61.8 s/349.8 °C).

It can be concluded that the heat equalization follows the same law as described above in Figure 8. The difference is basically an increase in the surface-to-volume ratio and a decrease in the average cross-section (thickness). 

We can find that the driving force of thermal equalization is greater in the case of a spindle with a larger surface-to-volume ratio and a smaller cross-section; therefore, thermal equalization occurs sooner. 

On the right side of Figure 15, it can be seen that heat flow reaches its highest speed at a temperature of 200 °C for both geometries.

With the spindle, this rate is significantly higher due to the advantages of a larger surface area. In this case, as temperature increases, the rate of heat flow (heat diffusion) decreases and stops around 1045 °C due to the thermal equalization. The difference is basically an increase in the surface-to-volume ratio and a decrease in the average cylindrical cross-section (thickness). 

Therefore, the driving force of heat equalization is greater in the case of a spindle with a larger surface-to-volume ratio and smaller cylindrical cross-section, thus heat equalization occurs sooner. It is also followed from Figure 15 that similar laws can be described both for surfaces and heating times. The first boundary condition is therefore fulfilled. Only a negligible surface of the spindle shape (near-lying state) is inactive; therefore, the entire surface can be considered active (shown in Figure 14). The cylinder was chosen as the theoretical base form for the study. Based on Equations (11) and (16), we can write:

(16) $$\frac{A}{V}\cdot x=\frac{t_{C}}{t_{S}}+\frac{A_{S}}{A_{CT}}$$

where $t_{C}$ is all heating time of the cylindrical-shaped specimen (s), $t_{S}\;$ is all heating time of the spindle-shaped specimen (s), $A_{S}\;$ is the total surface of the spindle-shaped specimen (m^2^) and $A_{CT}\;$ is the total surface of the cylindrical specimen (m^2^).

The usage of the entire surface in connection with Equation (14) requires an explanation. We had to take the entire surface into account, since in this way, we were able to make our analysis position-neutral. After that, substituting into Equation (14), the following heating time is obtained: 338.1 s. The obtained result is surprisingly close to the measured value of 339 s. 

Naturally, this method still needs to be refined with more data; however, we believe that it is basically suitable for direct application and further development in this research area. Potentially, artificial neural network models may be used to describe the relations among properties, even in the case of relatively small datasets [26].

## 5. Conclusions

In this article, the effect of shape dependence on heat transfer processes in the case of D20 mm and 20.75 × 20.75 mm cross-sectioned specimens with the same surface-to-volume ratio was reported. These specimens were made from S460N steel. 

Based on experience-drawn conclusions, we summarized the essential results, which are as follows:

- Complex geometric shapes can be characterized by a larger surface area and a lower heat transfer coefficient, which can lead to a shorter heating time in the case of the same surface-to-volume ratio.
- Heating time is basically determined by the size and shape of the active surfaces; therefore, in the case of the same shape, volume and total surface, the larger active surface can be associated with a shorter heating time of up to 20%.
- As a result of the experiments, a shape factor between the cylinder and the prismatic-shaped specimen was established, which equals 1.125.
- The mathematical relationship between the amount of heat that can be stored in the body during heat equalization and the complexity of the shape is established, which is characterized through ratios depending on heating times and active surfaces in the function of total surface-to-volume ratio.
- The mathematical relationship between the rate of heat equalization and the surface-to-volume ratio is established. These results point out that in the case of a larger surface-to-volume ratio, which means a smaller cross-section, heat equalization occurs sooner; consequently, the heating time will be shorter.

Overall, it can be said that our objective was achieved by creating relationships between shape complexity, heat transfer factors and heating time. Our outmost goal, to provide a more accurate method to estimate heating time, was also accomplished. 

## Figures and Tables

**Table 1:** Parameters and inputs for FEA modeling.

Parameters	Input
FEM software	Simufact Forming 15.0
Simulation type	2D
Cylinder geometry	Ø20 × 60 mm
Material model	S460
Workpiece initial temperature	25 °C
Heat transfer coefficient on average value	299.3 W/m^2^ °C
Emissivity of heat radiation for cold workpiece	automatic
Mesher type	2D-FE Quadtree
Element edge length	0.8 mm
Heating time	483.4 s
Furnace temperature	1046.5 °C

## Data Availability

The original contributions presented in the study are included in the article, further inquiries can be directed to the corresponding author.

## Abbreviations

Bi	the dimensionless Biot number (-)
$\alpha_{EHTC}$	the effective heat transfer coefficient (sum up convection and radiation) (W/m^2^·K)
$\kappa_{E}$	the effective thermal conductivity of scaled specimen (W/m·K)
$L^{\prime }$	the characteristic length (ratio of the volume and the surface) (m)
$\alpha_{R}$	the radiation heat transfer coefficient (W/m^2^·K)
$\alpha_{C}$	the convective heat transfer coefficient (W/m^2^·K)
ε	dimensionless emissivity constant (henceforth, its value is 0.8)
σ	the Stefan–Boltzmann constant (5.67 × 10^−8^ W/m^2^·K^4^)
$T_{S}$	the surface temperature of the specimen (K)
$T_{f}$	the furnace space temperature inside (K)
$\alpha_{R}$	the radiation heat transfer coefficient (W/m^2^·K)
Nu	the dimensionless Nusselt number (-)
L	the crucial length of the specimen (m)
$\kappa_{air}$	the effective thermal conductivity of the air film near the surface (W/m·K)
Ra	the dimensionless Rayleigh number (-)
D	the diameter of the specimen (m)
Pr	the dimensionless Prandtl number (-)
μ	the dynamic viscosity of dry air near the specimen surface (kg/m·s)
$c_{p(air)}$	the specific heat of dry air near the specimen surface (J/kg·K)
$\kappa_{air}$	the thermal conductivity of dry air near the specimen surface (W/m·K)
Gr	the dimensionless Grashof number (-)
g	the gravitational acceleration (9.81 m/s^2^)
β	the dry air thermal expansion coefficient a good approximation for gases is the reciprocal of the air temperature in Kelvin: (1/K)
$T_{air}$	the dry air film temperature that surrounds the specimen, which is the average of the surface temperature of the furnace space and test specimen (K)
ν	the kinematic viscosity of dry air near the specimen surface (m^2^/s)
X	the scale thickness at a given moment (μm)
R	the universal gas constant (8.314 J/K·mol)
T	the heating temperature which is equal to the surface temperature (K)
t	the heating time of specimen (s)
$c_{p}$	the specific heat of the specimen considering the scaled surface at 1045 °C (611.585 J/kg °C)
p	the density of the specimen considering the scaled surface at 1045 °C (7481.43 kg/m^3^)
x	the perpendicular distance between the surface and the core referring to the radius of cylinder (m)
λ	the heat conduction of S460N steel at 1050 °C (−28.35 W/m °C)
$\alpha_{EHTC(c)}$	the aggregated heat transfer coefficient of the cylindrical specimen at 1045 °C (427.5 W/m^2^ °C)
A/V	the value of the entire surface-to-volume ratio expressed in meters (m^−1^)
$t_{C}$	all heating time of the cylindrical-shaped specimen (s)
$t_{P}$	all heating time of the prismatic-shaped specimen (s)
$A_{P}$	the active surface of the prismatic-shaped specimen (m^2^)
$A_{C}$	the active surface of the cylindrical specimen (m^2^)
$t_{PN}$	all heating time of the new prismatic-shaped specimen (s)
$A_{PN}$	the active surface of the new prismatic-shaped specimen (m^2^)
a	the side distance of prismatic specimen (m)
H	the height of the specimen (m)
$D_{average}$	the average diameter of the specimen (m)
C	the heat capacity matrix (J/K)
$\dot{\vec{T}}$	the time derivative of the temperature (K/s)
K	the thermal conductivity matrix (W/m·K)
$\vec{T}$	the nodal temperature vector (m·K)
$\vec{Q}$	the thermal load vector (W)
$t_{S}$	all heating time of the spindle-shaped specimen (s)
$A_{CT}$	the total surface of the cylindrical specimen (m^2^)
$A_{S}$	the total surface of the spindle-shaped specimen (m^2^)
